# Concurrent partnerships in Cape Town, South Africa: race and sex differences in prevalence and duration of overlap

**DOI:** 10.7448/IAS.18.1.19372

**Published:** 2015-02-18

**Authors:** Roxanne Beauclair, Niel Hens, Wim Delva

**Affiliations:** 1 The South African Department of Science and Technology/National Research Foundation (DST/NRF) Centre of Excellence in Epidemiological Modelling and Analysis (SACEMA), Stellenbosch University, Stellenbosch, South Africa; 2 International Centre for Reproductive Health, Ghent University, Gent, Belgium; 3 Center for Statistics, Hasselt University, Diepenbeek, Belgium; 4 Centre for Health Economic Research and Modeling Infectious Diseases, Vaccine and Infectious Disease Institute, University of Antwerp, Wilrijk, Belgium

**Keywords:** concurrent partnerships, sexual concurrency, HIV prevention, South Africa, sexual behaviour and HIV, sexual risk behaviour

## Abstract

**Introduction:**

Concurrent partnerships (CPs) have been suggested as a risk factor for transmitting HIV, but their impact on the epidemic depends upon how prevalent they are in populations, the average number of CPs an individual has and the length of time they overlap. However, estimates of prevalence of CPs in Southern Africa vary widely, and the duration of overlap in these relationships is poorly documented. We aim to characterize concurrency in a more accurate and complete manner, using data from three disadvantaged communities of Cape Town, South Africa.

**Methods:**

We conducted a sexual behaviour survey (*n*=878) from June 2011 to February 2012 in Cape Town, using Audio Computer-Assisted Self-Interviewing to collect sexual relationship histories on partners in the past year. Using the beginning and end dates for the partnerships, we calculated the point prevalence, the cumulative prevalence and the incidence rate of CPs, as well as the duration of overlap for relationships begun in the previous year. Linear and binomial regression models were used to quantify race (black vs. coloured) and sex differences in the duration of overlap and relative risk of having CPs in the past year.

**Results:**

The overall point prevalence of CPs six months before the survey was 8.4%: 13.4% for black men, 1.9% for coloured men, 7.8% black women and 5.6% for coloured women. The median duration of overlap in CPs was 7.5 weeks. Women had less risk of CPs in the previous year than men (RR 0.43; 95% CI: 0.32–0.57) and black participants were more at risk than coloured participants (RR 1.86; 95% CI: 1.17–2.97).

**Conclusions:**

Our results indicate that in this population the prevalence of CPs is relatively high and is characterized by overlaps of long duration, implying there may be opportunities for HIV to be transmitted to concurrent partners.

## Introduction

Concurrent partnerships (CPs) have been identified as a potentially important facilitator of the HIV epidemic in Southern Africa [1–5]. CPs have been defined as “overlapping sexual partnerships in which sexual intercourse with one partner occurs between two acts of intercourse with another partner” [6]. Theoretically, CPs facilitate faster spread of HIV because of two different effects at the sexual network level: 1) earlier partnerships begun by the index partner are later exposed to any infections transmitted by an additional partner and 2) the time to secondary transmission is shortened because the infected person does not need to terminate one partnership before starting another. Proponents of this hypothesis have demonstrated through modelling studies that the effect of CPs on HIV transmission is large when high infectiousness during the acute stage of infection is taken into account [1,3,5]. Evidence from ecological studies has also shown that prevalence of HIV is correlated with prevalence of CPs [2,4,7,8].

In contrast with that evidence, data from rural KwaZulu-Natal, South Africa, indicated that women living in communities with high prevalence of CPs among men did not have increased risk of acquiring HIV [9]. Furthermore, a randomized controlled trial in Kisumu, Kenya, found that being infected with HIV at baseline was not associated with having CPs [10]. Reniers and Watkins proposed the coital dilution hypothesis as an explanation for why CPs are not likely to drive the spread of HIV [11]. They assert that as individuals acquire additional CPs, the average sex frequency per partner drops. Therefore, if the total number of partnerships in a population remains constant and the average coital frequency in those partnerships falls, then the total number of sex acts in the population must fall and result in a slower spread of HIV [11,12]. However, support for this theory has been mixed [13,14].

Go *et al*. argue that in order for CPs to be a major driver of HIV epidemics, three requirements must be met: first, the prevalence of CPs in a population must be high; second, the number of CPs should be moderately high for the average individual; and third, the duration of overlap should be relatively long [15]. It is currently difficult to tell if these conditions have been met in studied populations because definitions and measurement of CPs to date have been problematic. Many studies define CPs as any report of extra-spousal relationships (or casual relationships if they report a main partner) in the preceding 12 months [2,4,16,17]. These definitions assume that all participants are having sex with their spousal/main partners before and after the extra-spousal/casual sex. Moreover, this “direct” approach to measuring and defining concurrency precludes the capturing of beginning and end dates, which has meant that some studies could not calculate point prevalence of CPs, nor the duration of relationships, much less the length of time the relationships were overlapping [18–21]. In other studies where the duration of relationships is known, the measurement of relationship dates was accurate only up to one month [13,22]. This means that those studies could only capture relationships that overlapped by one month or more, missing potentially many more short-term concurrent relationships in their prevalence estimates. Still, some studies use better definitions of CPs but questions about CPs are asked in face-to-face-interviews [9,23,24], which may promote considerable social desirability bias [25] and underestimate the true prevalence of CPs.

There have been calls for a more refined definition of CPs and guidelines for measuring it [26]. The UNAIDS-proposed indicator of CPs is the point prevalence of having more than one sexual partnership, six months before the interview [6]. We believe this indicator may only be part of the solution. Even if point prevalence of CPs is calculated, further steps to improve accuracy must be taken, as well as combining this indicator with other measures of concurrency in order to produce a more complete and useful picture of CP dynamics. Not only is a more complete description of CPs important for an intuitive understanding of how HIV spreads in a population but this would also allow for better parameterization of epidemiological models that are used to study HIV (combination) prevention and HIV transmission dynamics.

In South Africa, where there are an estimated 6.4 million people currently living with HIV [27], it is important to obtain accurate estimates of CP point prevalence and the duration of overlapping relationships in order to understand why the prevalence of HIV is so high. Furthermore, race and sex are primary determinants of social, health, economic and educational opportunities in South Africa [28], and thus the relationship between them and CPs needs to be evaluated to determine which groups should be targeted for potential CPs and HIV risk-reduction interventions. There have been studies in Cape Town, South Africa, that have examined how race and sex are related to CPs [18,29–31]; however, none investigated the duration of overlap or incidence of entering a CP.

With this in mind, we analyzed data from a cross-sectional survey conducted in urban communities of Cape Town with a high prevalence of HIV. The survey questions were administered using Audio Computer-Assisted Self-Interviewing (ACASI), so as to minimize social desirability bias. We characterized concurrency in this population by estimating the point prevalence, cumulative prevalence, incidence and degree distribution of CPs. We also described the duration of overlaps for relationships begun in the previous year and the relative risk of having CPs for different race and sex groups.

## Methods

### Study design and setting

We conducted a cross-sectional survey (*n*=878) from June 2011 to February 2012 in three urban communities of Cape Town with high prevalence of HIV. The study communities were characterized by high unemployment, informal housing and very little post-secondary education [32]. These communities primarily have residents that identify as black or coloured. The participants were randomly sampled from a previous community randomized trial that aimed to reduce the prevalence of TB and HIV using novel public health interventions, the features of which have been previously published [33].

The survey was administered in a mobile office space on touchscreen computers. ACASI was used to provide participants with privacy while answering sensitive questions about their sexual behaviours. Participants could indicate on a visual timeline the beginning and end dates of relationships for up to five main partners and ten casual partners that they had in the previous 12 months. The dates were accurate up to one week within a month. Additional questions about condom use, age of partners, alcohol and drug use at first sex, sex frequency and proximity to partners were asked for relationships. The study was approved by the Stellenbosch University Health Research Ethics Committee (N11/03/093) and written, informed consent was obtained from each participant. Further details of our sampling strategy and study design have been published elsewhere [14,25,34].

### Participants

The survey had a contact rate of 60% (*n*=1115) for individuals enumerated in the sample frame, and a response rate of 85.4% (*n*=878) for those we contacted. We excluded participants from the analysis who were not between the ages of 15 and 70, or who had missing ages (*n*=34); had missing sex information (*n*=30); were not heterosexual (*n*=50); and who did not identify as black or coloured race, or had missing race information (*n*=14). We chose to include only the racial groups that have been previously characterized as having high HIV prevalence in their populations. The term “coloured” is an official racial category in South Africa, and is used to describe people who have racially mixed ancestry from Europe, indigenous populations, and South and East Asia. After exclusions, 750 participants remained and they reported on 1003 relationships. Of the 750 remaining participants, 148 did not report on any partner in the previous year. Characteristics of those participants can be found in the Supplementary file.

### Statistical analysis

All statistical analyses were performed using Stata statistical software, version 12.0 (Stata-Corp Inc., College Station, TX, USA). First, we calculated frequency distributions and summary statistics of relationship characteristics for participants (*n*=602) that reported having had at least one relationship in the past year (*n*=1003 relationships). Some relationship questions were only asked for relationships that started in the previous 12 months (*n*=415 relationships).

Next, by sex and race, we estimated the one-year cumulative prevalence, point prevalence six months before the survey and incidence rate of CPs. We calculated these in two ways: 1) all participants were included in the denominator (*n*=750), which is the indicator recommended by UNAIDS [6] and 2) only participants who reported having sex in the past year were in the denominator (*n*=528). Two relationships were categorized as concurrent if the beginning date of one relationship overlapped with the end date of the other relationship by at least one week. We expressed the incidence rate as number of cases per 1000 person-years, and calculated it by dividing the total number of CPs that occurred in the previous year by the total person-years spent at risk and then multiplying by 1000. Each person contributed one year of person-time to the denominator, because each day of the previous year provided another opportunity for a person to begin a new concurrent relationship. Central to our calculation of the incidence of CPs is the notion that a “case” is the occurrence of a concurrent relationship, not the event of an individual entering a new relationship while already engaged in an earlier relationship.

Next, for sexually active participants, we computed the median and range of the total number of partners in the previous year (regardless of whether they were concurrent or serially monogamous) and the total number of CPs. These same data are also presented as a frequency distribution for total numbers of partners (1/2/>=3) and CPs (0/2/>=3) in the past year. It is important to note here that it is not possible to have only one CP. At the moment when one of the relationships begins to overlap with another, they both become CPs, and thus the number of CPs will equal two. We calculated average duration of overlap for each relationship because each relationship could potentially overlap with more than one other relationship. Therefore, we present the median of participants’ average duration of overlaps, by sex and race. In this calculation, we excluded relationships that began outside of the one-year window because we do not have information on their complete durations.

In addition, we constructed univariate and multivariable binomial regression models with a log link function, in order to examine the risk of having a CP in the past 12 months for participants in different race-sex groups. Finally, we used simple and multiple linear regression models to look at the effect of race and sex on the duration of overlap for CPs begun in the previous year. Here, the unit of analysis was the relationship, and because each participant could report on more than one relationship, we used clustered sandwich estimators. For this sub-analysis, we used only relationships that began in the previous year (*n*=301), because we did not have complete durations for relationships that began outside of the one-year window. For each of the above regression analyses, a multivariable model was fit that included a race–sex interaction term, in order to see if race modifies the effect of sex.

## Results

Among participants who reported at least one relationship in the previous year, 20.9% (*n*=126) were coloured and 79.1% (*n*=476) were black; 32.4% (*n*=195) were male and 67.6% (*n*=407) were female. Characteristics of their relationships are presented in Table 1. Overall, most relationships reported by participants were from self-described “main” partnerships (76.1%) and the age difference between female and male partners tended to be less than five years (57.3%). Of note, black females had the highest percentage of age-disparate relationships (41.5%) and this demographic group had the highest proportion believing that their partner had other partners (58.6%). Coloured men had the highest proportion of casual relationships (42.3%) and non-condom use at the last sex (59.4%). Coloured women reported the highest percentage of relationships where the partner lived in the same home (34.6%) and the lowest fraction of using a condom at last sex (26.9%). Black men had the lowest median age of partner (26 years).

**Table 1:** Relationship characteristics of participants by race and sex

	Relationships (*n*=1003)				

		Men		Women	
	
		Black *n*=362	Coloured *n*=71	Black *n*=458	Coloured *n*=112
Age of partner, median (IQR)	32 (25–42)	26 (21–37)	37 (28–49)	34 (27–44)	34 (27–45)
Age disparate, *n* (%)					
Non-age disparate	575 (57.3)	201 (55.5)	48 (67.6)	250 (54.6)	76 (67.9)
Age disparate^a^	353 (35.2)	118 (32.6)	16 (22.5)	190 (41.5)	29 (25.9)
Missing	75 (7.5)	43 (11.9)	7 (9.9)	18 (3.9)	7 (6.3)
Partner type, *n* (%)					
Main	763 (76.1)	226 (62.4)	41 (57.8)	407 (88.9)	89 (79.5)
Casual	240 (23.9)	136 (37.6)	30 (42.3)	51 (11.1)	23 (20.5)
Still ongoing, *n* (%)					
Yes	617 (61.5)	173 (47.8)	33 (46.5)	323 (70.5)	88 (78.6)
No	364 (36.3)	181 (50.0)	33 (46.5)	126 (27.5)	24 (21.4)
I do not know	22 (2.2)	8 (2.2)	5 (7.0)	9 (2.0)	0 (0.0)
	**Relationships started in the past year (*n*=415)**				

		**Men**		**Women**	
	
		**Black *n*=217**	**Coloured *n*=32**	**Black *n*=140**	**Coloured *n*=26**

Where does your partner live in proximity to you?, *n* (%)					
Same house	68 (16.4)	30 (13.8)	7 (21.9)	22 (15.7)	9 (34.6)
Different house, same township	178 (42.9)	99 (45.6)	14 (43.8)	62 (44.3)	3 (11.5)
Different township, Western Cape	88 (21.2)	40 (18.4)	8 (25.0)	33 (23.6)	7 (26.9)
Different province, South Africa	40 (9.6)	23 (10.6)	1 (3.1)	13 (9.3)	3 (11.5)
I do not know/missing	41 (9.9)	25 (11.5)	2 (6.3)	10 (7.1)	4 (15.4)
Partner had other partners, *n* (%)					
Yes	188 (45.3)	88 (40.6)	14 (43.8)	82 (58.6)	4 (15.4)
No	168 (40.5)	90 (41.5)	16 (50.0)	44 (31.4)	18 (69.2)
I do not know/missing	59 (14.2)	39 (18.0)	2 (6.25)	14 (10.0)	4 (15.4)
Condom at last sex with partner, *n* (%)					
Yes	195 (47.0)	105 (48.4)	11 (34.4)	72 (51.4)	7 (26.9)
No	180 (43.4)	88 (40.6)	19 (59.4)	58 (41.4)	15 (57.7)
I do not know/missing	40 (9.6)	24 (11.1)	2 (6.3)	10 (7.1)	4 (15.4)
On alcohol at first sex, *n* (%)					
Yes	111 (26.8)	69 (31.8)	12 (37.5)	23 (16.4)	7 (26.9)
No	265 (63.9)	126 (58.1)	18 (56.3)	106 (75.7)	15 (57.7)
I do not know/missing	39 (9.4)	22 (10.1)	2 (6.3)	11 (7.9)	4 (15.4)

a Greater than five years difference between the female partner and the male partner.

IQR, inter-quartile range.

Estimates of prevalence, incidence rates and median duration of CPs overlap are presented in Table 2. Among all participants, the point prevalence of CPs was 8.4% (*n*=63), the one-year cumulative prevalence was 18.4% (*n*=138) and the incidence rate was 557.3 concurrent partners per 1000 person-years. The median duration of overlap for participants with CPs begun in the previous year was 7.5 weeks (IQR: 2.2–24), with coloured women having the longest duration of overlap at 26.5 weeks (IQR: 16–32). Black men had the highest point prevalence (13.4%), cumulative prevalence (34.2%) and incidence (1117.7 per 1000 person-years), compared to the other subgroups. The maximum number of relationships anyone had in the previous year was 12, with 29.5% of sexually active participants having more than one relationship. Overall, 11.7% of sexually active participants had three or more CPs in the previous year. Men of both races tended to have more CPs than women, and for both sexes, black participants had more CPs than coloured participants. For both black and coloured men, there was a higher frequency of three or more CPs (25.9 and 12.1%, respectively) than just two CPs (20.0 and 9.1%, respectively). Overall, of the 156 participants who had two or more relationships in the past year, 85.9% (*n*=134) had concurrent as opposed to serially monogamous partnerships. In all of the race–sex subgroups except coloured women, the cumulative prevalence of CPs was substantially higher than the estimated point prevalence. In particular, among coloured men, the cumulative prevalence (13.0%) was nearly seven times higher than the point prevalence (1.9%). This difference is most likely because of the relatively high incidence of CPs (500 per 1000 person-years) and short median duration of overlaps (four weeks).

**Table 2:** Estimates of concurrent relationships by race and sex

	Total	Men		Women	
	
		Black	Coloured	Black	Coloured
**Six months before survey**					
Concurrency among all participants, *n* (%)	63 (8.4)	25 (13.4)	1 (1.9)	30 (7.8)	7 (5.6)
Concurrency among sexually active participants, *n* (%)	61 (11.6)	24 (17.8)	1 (3.0)	29 (10.1)	7 (9.7)
**Any time in the past year**					
Among all participants					
Concurrency, *n* (%)	138 (18.4)	64 (34.2)	7 (13.0)	58 (15.1)	9 (7.1)
Incidence rate of concurrency,	557.33	1117.65	500.00	420.37	166.67
*n* per 1000 person-years (95% CI)	(505.18–613.41)	(971.25–1279.88)	(329.50–727.47)	(357.94–490.55)	(103.17–254.77)
Among only sexually active participants					
Concurrency, *n* (%)	134 (25.4)	62 (45.9)	7 (21.2)	56 (19.4)	9 (12.5)
Incidence rate of concurrency,	767.05	1481.48	818.18	545.14	291.67
*n* per 1000 person-years (95% CI)	(694.15–845.51)	(1283.27–1701.64)	(539.19–1190.41)	(463.20–637.39)	(180.55–445.84)
Total number of relationships, med (Range)	1 (1–12)	1 (1–11)	1 (1–12)	1 (1–11)	1 (1–6)
**Total number of relationships, *n* (%)**					
1	372 (70.5)	66 (48.9)	23 (69.7)	223 (77.4)	60 (83.3)
2	66 (12.5)	21 (15.6)	2 (6.1)	36 (12.5)	7 (9.7)
≥3	90 (17.0)	48 (35.6)	8 (24.2)	29 (10.1)	5 (6.9)
Number of concurrent relationships, med (Range)	0 (0–11)	0 (0–10)	0 (0–11)	0 (0–10)	0 (0–3)
**Number of concurrent relationships, *n* (%)**					
0	394 (74.6)	73 (54.1)	26 (78.8)	232 (80.6)	63 (87.5)
2	72 (13.6)	27 (20.0)	3 (9.1)	36 (12.5)	6 (8.3)
≥3	62 (11.7)	35 (25.9)	4 (12.1)	20 (6.9)	3 (4.2)
Duration of overlap (weeks), med (IQR)	7.5 (2.2–24)	6.75 (2–17)	4 (1–7.6)	9.4 (2–43)	26.5 (16–32)

IQR, inter-quartile range; CI, confidence interval.

The results from the binomial regression analysis can be found in Table 3. Both the crude and adjusted risk ratios indicate that women were at a lower risk than men of having had CPs in the previous 12 months (aRR 0.43; 95% CI: 0.32–0.57). In the adjusted model, black people were almost twice as likely to have had CPs (aRR 1.86; 95% CI: 1.17–2.97). The interaction term added to the multivariable model was not significant. The results in Table 4 show the effect of sex and race on the duration of overlap for 301 concurrent relationships belonging to 133 people. There is a significant effect of sex and race on duration of overlap when an interaction term is included. In coloured participants, the average duration of overlap was 19.43 weeks longer for females than males (95% CI: 7.09–31.76). The average duration of overlap for relationships begun in the previous year was 8.1 weeks longer for black men compared to coloured men (95% CI: 2.6–13.6).

**Table 3:** Binomial regression models for relative risk of concurrency in race–sex groups

	Model 1 Crude RR (95% CI)	Model 2 Crude RR (95% CI)	Model 3 Adjusted RR (95% CI)	Model 4 Adjusted RR (95% CI)
Sex				
Male	1.00	–	1.00	1.00
Female	0.43 (0.32–0.57)		0.43 (0.32–0.57)	0.57 (0.23–1.40)
Race				
Coloured	–	1.00	1.00	1.00
Black	–	1.85 (1.15–2.97)	1.86 (1.17–2.97)	2.16 (1.10–4.27)
Race-sex interaction	–	–	–	0.73 (0.28–1.87)

RR, risk ratio; CI, confidence interval.

**Table 4:** Estimates for the effect of sex and race on duration of overlap in concurrent relationships that began in the previous year

	Model 1 Crude β-coefficient (95% CI)	Model 2 Crude β-coefficient (95% CI)	Model 3 Adjusted β-coefficient (95% CI)	Model 4 Adjusted β-coefficient (95% CI)
Intercept	12.99 (8.68–17.31)	14.07 (6.87–21.26)	12.20 (5.70–18.70)	5.89 (3.31–8.47)
Sex				
Female	4.42 (−1.88–10.72)	–	4.43 (−1.87–10.73)	19.43 (7.09–31.76)
Race				
Black	–	0.81 (−7.17–8.78)	0.90 (−6.33–8.13)	8.10 (2.63–13.56)
Race–sex interaction	–	–	–	−17.20 (−31.30–3.10)

CI, confidence interval.

## Discussion

Using data from our sexual behaviour survey located in disadvantaged communities of Cape Town, we aimed to estimate and describe CPs in different race and sex groups using a nuanced and holistic definition of CPs. The results of our analysis indicate that not only is there a high incidence and prevalence of CPs in the study communities but the duration of overlapping relationships is also long. Indeed, among all participants we observed a point prevalence of 8.4%, cumulative prevalence of 18.4% and a 7.5-week median duration of overlap among those who had CPs. Importantly, our data also show that most people with two or more relationships in the previous year did not have serially monogamous relationships.

Specifically, among sexually active participants, we see that 45.9% of black men, 19.4% of black women, 21.2% of coloured men and 12.5% of coloured women were engaged in CPs during the past year. This is comparable to the 2009 CP prevalence estimates of Maughan-Brown in his analysis of sexually experienced participants in the Cape Area Panel Study (CAPS): 39% of black men, 14% of black women, 8% of coloured men and 1% of coloured women [30]. Both our study and the CAPS data indicate that black men have the highest frequencies of engaging in CPs. Overall, our study produced higher estimates for all race–sex groups. This may be due to differences in several factors including, but not limited to: the specific areas under study, how the denominators were calculated, how CPs were measured and the mode of interviewing.

Crucially, we see that black men had the highest cumulative prevalence, point prevalence and incidence rates compared to the other three demographics. Black men had 2.16 (95% CI: 1.10–4.27) times the risk of having CPs in the previous year compared to coloured men. More than half of the black men who reported CPs had three or more CPs in the previous year. Kenyon *et al*. report that black people in South Africa have more favourable attitudes towards concurrency than coloured and white people, and this may explain the reasons why prevalence is highest in this group [35]. It has been previously noted that if a specific subgroup of a population is engaging in greater numbers of CPs, such as black men in our population, then the connectedness of the sexual network that they are part of also increases, which may thus increase their HIV transmission probability in a non-linear fashion [31,36].

In addition, all of the race–sex groups investigated in our study have CPs that are characterized by median durations of overlap of four weeks or more. Coloured women had the longest overlaps with the median duration lasting more than six months. This observation is consistent with the finding that coloured women had higher point prevalence but not higher cumulative prevalence of CPs than coloured men. Under the assumption that most coloured women choose coloured men for partners and that it may be easier to hide a concurrent relationship of short duration, the longer overlaps in coloured women versus men may also explain the finding that coloured men were more likely to think that their partners had other partners.

Whether or not to classify the observed durations of overlap as long, is a subjective judgement call. Perhaps the most objective and relevant reference point is the duration of the acute phase of HIV infection. Estimates for the duration of this phase range from one to three months [37,38]. In light of this point of reference, the median durations of overlap observed in our survey are relatively long. The epidemiological implication of long overlaps in relationships is that they may give rise to stable, connected sexual networks because participants go back and forth between sexual partners over the course of several weeks to months. This means that a person's risk of HIV acquisition will be influenced by the behaviour of others in the network, in addition to his or her own behaviour [39]. These relationships are also more likely to take full advantage of the high viral load and associated high infectiousness during the acute phase of infection, with enhanced transmission potential as a result.

Another key idea that our analysis elucidates is that it is not just men who have high frequencies of engaging in CPs. Large fractions of women also had CPs during the previous year: 19.4% and 12.5% black and coloured sexually active women, respectively. Indeed, out of 65 black women who reported more than one relationship in the previous year, all but nine engaged in concurrency, as opposed to serial monogamy. Many previous studies have reported relatively smaller proportions of women engaging in CPs [30,40,41]. Still, other studies that have investigated the relationship between HIV and CPs have neglected to incorporate CPs from women in their estimates altogether [7,9,10,17]. We argue that in order to understand how or if CPs affects HIV transmission, it is essential to also have accurate estimates of CPs in women and include their contributions to making the sexual network more connected.

Our study has several implications for how research related to estimating the occurrence of CPs is conducted. First, it gives insight into the utility of the UNAIDS proposed indicator of measuring the point prevalence of CPs six months before the survey. As expected, based on the relationship overlap data, we found large differences between the reported point prevalence and cumulative prevalence, especially for men. Men in our study had shorter durations of overlap and so cross-sectional snapshots of the sexual network (i.e. point prevalence estimates) were less likely to include the short concurrent relationships. This implies that the point prevalence alone may not be a sufficient proxy for studies investigating the relationship between HIV and CPs. Linking the cumulative prevalence indicator to the point prevalence indicator and duration of overlap can help to distinguish between different types of CP patterns at the population level. For example, if point prevalence and cumulative prevalence are small, and the average duration of overlap is short, relative to the period of observation, this would suggest that engaging in CPs is confined to a small subgroup of individuals. Large differences between point prevalence and cumulative prevalence, despite relatively short mean duration of overlap, suggest that there is a large fraction of people infrequently engaging in CPs.

Measuring the incidence of CPs may add value to studies that estimate the effect of behavioural change interventions. For instance, there may be settings in which a small core group of individuals is engaging in a high number of short CPs each year. If the intervention reduces the rate of acquiring CPs, but not the size of this core group, then the one-year cumulative prevalence of CPs before and after the intervention would be similar. Moreover, the short duration of overlap would result in point prevalence estimates of CPs that are very small, and therefore may lack statistical power to detect differences over time. Our study suggests that point prevalence, incidence, cumulative prevalence, number of relationships and duration of overlap, in combination, tell a more complete story. High proportions of people engaging in CPs (i.e. cumulative prevalence), high frequency of engaging in CPs (i.e. incidence) and long overlaps would lead to a highly connected network and faster spread of HIV.

Second, our study offers a better way to measure concurrency for future studies that investigate the relationship between CPs and HIV [42]. Although UNAIDS suggests an indicator that researchers should use, it does not elaborate on how the source data should be collected. The largest strength of our study is that our estimates of CPs were derived from a questionnaire that used ACASI, which has been shown to increase reporting of sensitive sexual behaviours in other African contexts [43–46] and reduce social desirability bias in our own study [25]. Furthermore, the questionnaire was presented on a touchscreen desktop computer, which exhibited a visual timeline with dates during the previous year. The durations for each relationship were constantly displayed below the timeline in different colours to help participants recall new relationships in the context of dates for previous relationships they enumerated. Visual timelines have been shown to foster internal consistency in reporting relationship dates [47,48]. Moreover, we believe our questionnaire reduced fatigue bias because it was designed specifically to ask relatively few questions about sexual behaviours in each relationship. This contrasts with DHSs, for example, which have been previously used to estimate CP prevalence in different populations [2,49]. Also, we believe our more accurate CPs estimates lend themselves well as calibration data for modelling studies of the spread of HIV and effectiveness of combination prevention strategies in an urban South African context.

Our study is not without limitations. First, our CPs estimates may still be biased despite using an ACASI questionnaire. A study conducted on Likoma Island in Lake Malawi, using socio-centric data to look at inter-partner agreement of reports of sexual relationships, indicated that CPs estimates may still be unreliable even when ACASI questionnaires are administered [50]. Furthermore, the same study also found that relationships of longer duration were more likely to be reported and short-term relationships may be underreported by participants. Date heaping is another form of bias that may be present, resulting in overestimates of concurrency [51]. However, we believe our study was less likely to show these forms of bias due to the visual timeline that we used to help people accurately recall their relationships and their durations. A second limitation of our study is that our data were left truncated, and therefore we do not have the beginning dates of relationships that started one year before the survey. This means that the median duration of overlap we calculated in Table 2 is probably an underestimate of the true median, because it is possible that two relationships that were overlapping at the start of the previous 12-month window, had been overlapping before the period of observation. The fact that the data were left truncated limited the number of relationships we could use for our analysis of the effect of sex and race on duration of overlap in CPs. It also prevented us from performing more advanced time-to-event analyses for the risk of entering CPs.

Although we do not have any evidence to advocate for or against CPs interventions, we believe that it is premature to give up on behavioural interventions as a mode for HIV prevention. The lack of evidence for the effectiveness of CPs-reduction interventions does not necessarily mean that such interventions are ineffective. Many reasons for this lack of evidence exist. In the current context of a strong international focus on biomedical interventions for HIV prevention, behavioural research is relatively underfunded [52]. As a result, studies with a primary focus on sexual behaviour lack funds to collect HIV biomarkers [53]. Alternatively, many studies with a primary focus on biomedical interventions include the measurement of HIV incidence but studies of sexual relationship histories are often treated as secondary analyses [54], and suboptimal designs for collecting this sensitive information are used.

## Conclusions

Although we cannot provide evidence of CPs influencing HIV transmission, we do offer a useful way forward for measuring and defining CPs for future studies. We have demonstrated that in economically disadvantaged areas around Cape Town, CPs rates are high and the average duration of overlaps are relatively long. The conditions needed to create and maintain a highly connected sexual network have been met in this setting. Our study does not lend itself to providing evidence for the commencement, continuation or termination of public health interventions related to CPs. However, our estimates may be useful in future modelling studies that attempt to improve our understanding of what combination of CPs incidence, prevalence and duration of overlap would be sufficient to result in sizeable increases in the rate of HIV transmission. We believe it would be useful to repeat our survey and analysis in other settings with varying degrees of HIV prevalence to see if further associations can be found with our proposed suite of CP indicators and HIV.

## Supplementary Material

Concurrent partnerships in Cape Town, South Africa: race and sex differences in prevalence and duration of overlap
