# Supplementary material for: Concurrent partnerships in Cape Town, South Africa: race and sex differences in prevalence and duration of overlap
Source: J Int AIDS Soc. 2015 Feb 18;18(1):19372. doi: 10.7448/IAS.18.1.19372 (PMC4334769; doi:10.7448/IAS.18.1.19372)
Supplement: Concurrent partnerships in Cape Town, South Africa: race and sex differences in prevalence and duration of overlap [file JIAS-18-19372-s001.pdf]

ADDITIONAL FILES

Additional file 1:

This is a word document table for socio-demographic characteristics of participants with and without relationships in the previous year. It should be included as a supplementary table.

Table 1: Socio-demographic characteristics for participants with and without relationships in the previous year

	Participants without relationships (n=148)	Participants with relationships (n=602)
**Age** med (IQR)	42 (29-52)	34 (26-44)
**Race**		
**Coloured**	54 (36.5)	126 (20.9)
**Black**	94 (63.5)	476 (79.1)
**Gender**		
**Male**	46 (31.1)	195 (32.4)
**Female**	102 (68.9)	407 (67.6)
**Employed**		
**No**	123 (83.1)	468 (77.7)
**Yes**	25 (16.9)	132 (21.9)
**Missing**	0 (0.0)	2 (0.3)
**Religion**		
**Christian**	106 (71.6)	395 (65.6)
**Not religious**	29 (19.6)	171 (28.4)
**Other**	13 (8.8)	36 (6.0)
**Highest Grade Completed**		
**None or Primary**	82 (55.4)	205 (34.1)
**Secondary**	64 (43.2)	375 (62.3)
**Tertiary**	1 (0.7)	20 (3.3)
**Missing**	1 (0.7)	2 (0.3)

IQR, Inter-quartile Range
